# Plasma Vitamin B12 May Be a Misleading Biomarker among Children with Severe Acute Malnutrition: An Observation from Mwanza, Tanzania

**DOI:** 10.1016/j.tjnut.2025.04.033

**Published:** 2025-05-16

**Authors:** Rikke Møller, Ebba Nexo, George PrayGod, Belinda Kweka, Happyness Kunzi, Maimuna Ahmed, Suzanne Filteau, Melissa Gladstone, André Briend, Henrik Friis, Mette Frahm Olsen

**Affiliations:** 1 Department of Infectious Diseases, Rigshospitalet, Copenhagen, Denmark; 2 Department of Clinical Biochemistry, Aarhus University Hospital, Aarhus, Denmark; 3 National Institute for Medical Research (NIMR), Muhimbili Centre, Dar es Salaam, Tanzania; 4 National Institute for Medical Research (NIMR), Mwanza Centre, Mwanza, Tanzania; 5 Catholic University of Health and Allied Sciences (CUHAS), Mwanza, Tanzania; 6 Department of Paediatrics, Bugando Medical Center, Mwanza, Tanzania; 7 Faculty of Epidemiology and Population Health, London School of Hygiene and Tropical Medicine (LSHTM), London, United Kingdom; 8 Department of Women and Children’s Health, University of Liverpool, Liverpool, United Kingdom; 9 Department of Nutrition, Exercise and Sports, University of Copenhagen, Copenhagen, Denmark; 10 Tampere Center for Child, Adolescent and Maternal Health Research, Faculty of Medicine and Health Technology, Tampere University, Tampere, Finland

**Keywords:** malnutrition, micronutrient deficiency, cobalamin, haptocorrin, nutritional treatment

## Abstract

**Background:**

Vitamin B12 deficiency is related to impaired neurodevelopment and anemia and is a global health concern in children with severe acute malnutrition (SAM).

**Objectives:**

We investigated vitamin B12 status in children with SAM and among non-malnourished comparisons and assessed the validity of the plasma vitamin B12 assay.

**Methods:**

This study was part of a pilot study of a nutritional intervention. We measured plasma concentrations of vitamin B12, methylmalonic acid (MMA), total haptocorrin (HC), and total transcobalamin (TC) in children with SAM before and after 8 wk of nutritional treatment and at a single timepoint in non-malnourished children.

**Results:**

We included 82 children with SAM and 82 children without acute malnutrition. Plasma vitamin B12 was higher in children with SAM at enrollment than in controls (median 647 compared with 411 pmol/L) and declined after treatment (median 469 pmol/L). Baseline plasma vitamin B12 was particularly high in children with edematous SAM compared to those without edema (*P* = 0.050). In contrast, plasma MMA was higher in those with SAM, reflecting lower vitamin B12 function (median 220 compared with 170 nmol/L), and declined after treatment (median 190 nmol). HC was positively correlated with vitamin B12 concentration (*P* < 0.001) and was also higher in children with SAM than in comparison children (median 959 compared with 789 pmol/L), decreasing after treatment (median 815 pmol/L). TC was higher in children with SAM than comparisons but did not decrease after treatment. Overall, 23% of children with SAM had MMA concentrations suggesting vitamin B12 deficiency compared to 4.5% of non-malnourished children.

**Conclusions:**

In contrast to our initial expectations, we found that plasma vitamin B12 was higher in children with SAM, especially those with edema. However, analysis of MMA revealed lower vitamin B12 function in malnourished children and improvement after treatment, warranting caution in the interpretation of plasma vitamin B12 in SAM. Elevated HC in SAM may explain high vitamin B12 concentration, but the cause of elevated HC needs further investigation.

## Introduction

Malnutrition among children remains a major global health concern. In 2022, ≥45 million children were affected by moderate or severe acute malnutrition (SAM), defined as a low weight-for-height *z*-score (WHZ <−2) [1]. This number reflects the estimated prevalence of wasted children at a given time; thus, the yearly incidence of acute malnutrition—including children with edematous malnutrition—is likely higher. These children generally lack various micronutrients [2], and there is now a growing recognition of the importance of vitamin B12 deficiency among children with malnutrition [3]. Animal-source foods constitute the primary dietary reservoir of vitamin B12, placing individuals in low-income settings at a heightened risk of deficiency [4,5]. Insufficient vitamin B12 concentrations have been linked to anemia and cognitive deficits in this vulnerable population [6]. A study from India suggested that supplementation with vitamin B12 may also improve linear and ponderal growth in malnourished children [7], possibly indirectly by reducing anemia [8]. Recent studies in children with acute malnutrition have found biomarker concentrations that suggest a high prevalence of vitamin B12 deficiency. In Burkina Faso, 63% of children with moderate acute malnutrition had marginal (149–222 pmol/L) or low (≤148 pmol/L) vitamin B12 concentrations [6], and 84% of children with SAM had a 3cB12 score, which is calculated from 3 different vitamin B12-related biomarkers, indicating deficiency [9].

As part of a pilot study of a nutritional intervention (the BrightSAM trial development study [10]), we measured plasma vitamin B12 before and after nutritional treatment among children with SAM and at a single time point in non-malnourished controls. Our aim was to describe the prevalence of vitamin B12 deficiency in our study setting and to inform the potential need for vitamin B12 supplementation in the subsequent trial. However, initial analysis of plasma vitamin B12 showed higher concentrations in children with SAM than in those without malnutrition. This observation led us to expand our objectives to include assessment of other biomarkers and discuss the validity of plasma vitamin B12 as a marker of vitamin B12 status in children with SAM.

We therefore added analyses of methylmalonic acid (MMA) as another marker of vitamin B12 status. Since methylmalonyl-CoA, the metabolically active form of MMA, is the substrate for a vitamin B12-dependent enzyme, an increase in MMA reflects a functional vitamin B12 deficiency [3]. We also included analyses of the vitamin B12 binding proteins transcobalamin (TC) and haptocorrin (HC) to help explain variations in plasma vitamin B12. Increases in these proteins generally lead to elevated plasma vitamin B12, and high measured vitamin B12 may therefore reflect increased binding proteins, notably HC, rather than vitamin B12 status in tissue [3,11].

In the present paper, we present concentrations of plasma vitamin B12, MMA, HC, and TC in children with SAM, measured before and after treatment, and compare them with concentrations in non-malnourished children. Based on this, we discuss the validity of plasma B12 as a biomarker of B12 status in this population.

## Methods

### Study design and setting

This study was performed as a part of the BrightSAM trial development study, which evaluated an 8-wk combined intervention of ready-to-use therapeutic food (RUTF) with a modified composition of poly unsaturated fatty acids (PUFAs) and a psychosocial intervention among children with SAM and compared their characteristics to a group of non-malnourished untreated children [10,12]. The study was conducted near Mwanza in the Lake Region, Tanzania, where children were included from 1 of 4 sites: Bugando Medical Centre, the referral hospital for the Lake Region, situated in Mwanza city; Misungwi District Hospital; Buzuruga Health Centre; and Nyamagana District Hospital. Community Health Workers connected to each site were trained in community screening using mid-upper arm circumference (MUAC) tapes and assessment of nutritional edema to identify SAM cases.

### Participants

We included children with SAM between 6 and 36 mo of age with a MUAC <115 mm and/or weight-for-height *z*-score (WHZ) <−3 and/or bipedal pitting edema. Children were enrolled into the study either directly after diagnosis of SAM, if they were eligible for outpatient care, or after initial inpatient stabilization. Criteria for inpatient care were clinical features of infections, bilateral pitting edema of any grade, or a failed appetite test [13]. Admitted children were enrolled in the study when they transitioned from liquid therapeutic diet (F-75) to RUTF. For comparison, we recruited children who did not have acute malnutrition (MUAC >125 mm and WHZ >−2) and were apparently well, frequency-matched by age (±2 mo) and sex, from communities in the same areas as the children with SAM. Children in both groups were excluded if they had severe disability that would hinder delivery of interventions or evaluation of outcomes or if they had known allergies to the contents of the nutrition intervention product, including peanuts. Informed consent from caregivers aged ≥18 y was collected for all participants.

### Nutritional intervention

Nutritional treatment was provided as daily portions of RUTF, distributed at weekly visits, for a total treatment duration of 8 wk. Dosage was weight dependent, increasing from 2, 3, and 4 sachets per day for a child weighing 5 to <7, 7 to <10, and 10 to <15 kg, respectively. The RUTF used in this study was a peanut butter-based paste with milk powder, whey, sugar, and vegetable fats, fortified with micronutrients and electrolytes (see Supplemental Table 1). This RUTF had a modified balance of n–3 to n–6 PUFA in favor of n–3 fatty acids, and the product contained 3.7 g n–6 fatty acids and 1.02 g n–3 fatty acids per 100 g. The mean amount of vitamin B12 per 100 g of RUTF was 1.6 μg.

### Sociodemographic and clinical data collection

All children were assessed by a study clinician at enrollment. Key clinical parameters including nutritional edema were registered, and caregivers were asked about HIV exposure and developmental delays. Sociodemographic data, including age, sex, parental education and occupation, and household assets and clinical data including birth weight were collected using standardized forms.

### Laboratory analyses

Plasma samples were collected at baseline (all participants) and after 8 wk of intervention (only children with SAM) in EDTA-plasma tubes. After sampling, tubes were stored at 2 to 8°C for a maximum of 8 h until arrival at the laboratory of the National Institute of Medical Research (NIMR) Mwanza Centre. There, samples were centrifuged, and plasma was stored at −80°C until it was transported on dry ice to Copenhagen University Hospital for analysis of vitamin B12 and to Aarhus University Hospital for analyses of MMA, HC, and TC.

Plasma vitamin B12 was measured using a commercial competitive electrochemiluminescence protein binding assay on a Cobas 8000, e801 module (Roche Diagnostics). Concentrations >1480 pmol/L were not further diluted and were assigned the maximum value. MMA was measured by liquid chromatography–tandem mass spectrometry on the AB SCIEX Triple Quad 5500 System (AB SCIEX LLC), and total HC and total TC were measured using in-house enzyme linked immunosorbent assays [14,15].

### Statistical analyses

Participant characteristics were summarized and presented as n (%) or mean ± SD. Socioeconomic status (SES) was described by computing principal component analysis of household assets, in which each SES group was defined by quintiles of the first principal component. Baseline data were compared between children with SAM and comparisons using Student’s t-test for continuous variables and chi-square test for categorical variables. Biomarkers of vitamin B12 status were summarized descriptively by median (IQR) and mean ± SD. Vitamin B12, MMA, and HC data were compared to available reference material for the relevant age group and categorized as normal, low, or high concentrations of each biomarker [16,17]. Reference levels were vitamin B12: 180 to 1400 pmol/L (0–12 mo) and 260 to 1200 pmol/L (1–12 y) [17]; HC: 370 to 1240 pmol/L (2–10 y) [16]; and MMA: 100 to 1250 nmol/L (0–12 mo) and 100 to 300 nmol/L (1–12 y) [17]. No age-matched reference was available for TC.

We compared biomarkers before and after treatment in children with SAM using paired t-tests. The biomarkers in children with SAM were compared to those in non-malnourished children using linear regression models to allow for adjustments of differences in age and sex. For vitamin B12 analyses, linear regression was based on Tobit models due to right-censoring of the data. MMA data were log transformed prior to analysis due to nonnormal distribution. In addition, we assessed correlations between vitamin B12 and other biomarkers using Tobit regression. Statistical analyses were performed in STATA 17 (StataCorp) or in R statistical software (v4.1.0) with R Studio (v1.2.5001).

### Ethics

The BrightSAM trial development study was approved by the Medical Research Coordinating Committee of the National Institute for Medical Research (NIMR), Tanzania (approval no. NIMR/HQ/R.8a/Vol.IX/3340) and the London School of Hygiene and Tropical Medicine, United Kingdom (approval no. 17831).

## Results

We included 82 children with SAM and 88 non-malnourished comparisons in the BrightSAM trial development study. Of these, 82 children from both groups had available data for ≥1 vitamin B12-related biomarker and were thus included in this study. Children with SAM tended to be younger than non-malnourished children, were more likely to be HIV-positive, and came from households with lower wealth indices and maternal education levels (Table 1). Five children with SAM died during the study, and 7 were lost to follow-up. Additional missing data were due to insufficient material for biochemical analyses. Thus, for baseline/follow-up/comparisons, results are reported from 78/66/80 children for vitamin B12 data; 73/65/76 children for HC data; 76/66/75 for TC data, and 78/56/67 for MMA data, respectively.

**TABLE 1:** Characteristics of children with SAM and no malnutrition.

	SAM (n = 82)	No malnutrition (n = 82)	*P*
**Sociodemographic characteristics**^1^			
Girls, *n* (%)	43 (52.4)	39 (47.6)	0.64
Age, mo (mean ± SD)	15.4 ± 6.8	17.7 ± 8.0	0.054
Parents’ marital status, *n* (%)			0.48
Married or cohabiting	47 (57.3)	54 (65.9)	
Divorced, separated, or widowed	13 (15.9)	12 (14.6)	
Never married	22 (26.8)	16 (19.5)	
Maternal education, *n* (%)			0.01
Never went to school	16 (20.3)	6 (7.8)	
Primary school	50 (63.3)	45 (58.4)	
Secondary school or higher^2^	13 (16.5)	26 (33.8)	
Maternal occupation, *n* (%)			<0.001
Salaried employment	3 (3.9)	1 (1.3)	
Petty trader (self-employed)	18 (23.4)	38 (50.0)	
Farmer (self-employed)	16 (20.8)	2 (2.6)	
Housewife/unemployed/student	40 (52.0)	35 (46.1)	
Household wealth index, quintile, *n* (%)			
Lowest	26 (31.7)	7 (8.5)	0.002
Second	17 (20.7)	16 (19.5)	
Third	16 (19.5)	17 (20.7)	
Fourth	12 (14.6)	20 (24.4)	
Highest	11 (13.4)	22 (26.8)	
**Developmental delay**^3^,n (%)			
Visual deficits	7 (8.5)	3 (3.7)	0.19
Hearing deficits	8 (9.8)	3 (3.7)	0.12
Delayed development	42 (51.2)	0 (0.0)	<0.001
**HIV exposure**^3^			
Child HIV-positive^4^, *n* (%)	7 (13.5)	0 (0.0)	<0.001
HIV-positive children on antiretrovirals, *n* (%)	6 (85.7)	NA	NA
Mother HIV-positive^4^, *n* (%)	17 (23.6)	1 (5.0)	<0.001

Abbreviations: NA, not applicable; SAM, severe acute malnutrition.

No malnutrition = mid-upper arm circumference (MUAC) >125 and weight-for-height z-score (WHZ) >−2. Data shown as n (%) or mean ± SD. *P* value for group difference based on Student t-test (continuous variables) and chi-squared test (categorical variables).

1 Data occasionally missing from non-malnourished comparisons.

2 Any or completed.

3 Reported by caregiver.

4 Percentage of participants with known HIV status number (SAM, *n* = 52 children and *n* = 71 mothers; no malnutrition, *n* = 20 children and *n* =76 mothers).

### Plasma concentrations of vitamin B12 and related biomarkers

At baseline, only 7.7% of children with SAM had low concentrations of vitamin B12, whereas MMA was elevated in 23.1% of children with SAM compared with 4.5% of non-malnourished children (Table 2). Plasma vitamin B12 was elevated in 9% of children with SAM but in none after treatment or in the comparison group. At baseline, HC was elevated in one-third of children with SAM; this proportion decreased to ∼10% after treatment, which was comparable to the comparison group.

**TABLE 2:** Distributions according to reference intervals of plasma vitamin B12, MMA, and HC in children with SAM and non-malnourished children.

	*n*	SAM baseline			*n*	SAM follow-up			*n*	No malnutrition		
		High (%)	Normal (%)	Low (%)		High (%)	Normal (%)	Low (%)		High (%)	Normal (%)	Low (%)
B12	78	9.0	83.3	7.7	66	0.0	90.9	9.1	80	0.0	97.5	2.5
MMA	78	23.1	71.8	5.1	56	16.1	73.2	10.7	67	4.5	80.6	14.9
HC	73	32.9	67.1	0.0	65	9.2	87.7	3.1	76	11.8	86.8	1.3

Abbreviations: HC, haptocorrin; MMA, methylmalonic acid; SAM, severe acute malnutrition.

Reference levels for B12: 180 to 1400 pmol/L (0–12 mo) and 260 to 1200 pmol/L (1–12 y); HC: 370 to 1240 pmol/L (2–10 y); MMA: 100 to 1250 nmol/L (0–12 mo) and 100 to 300 nmol/L (1–12 y).

**FIGURE 1:**
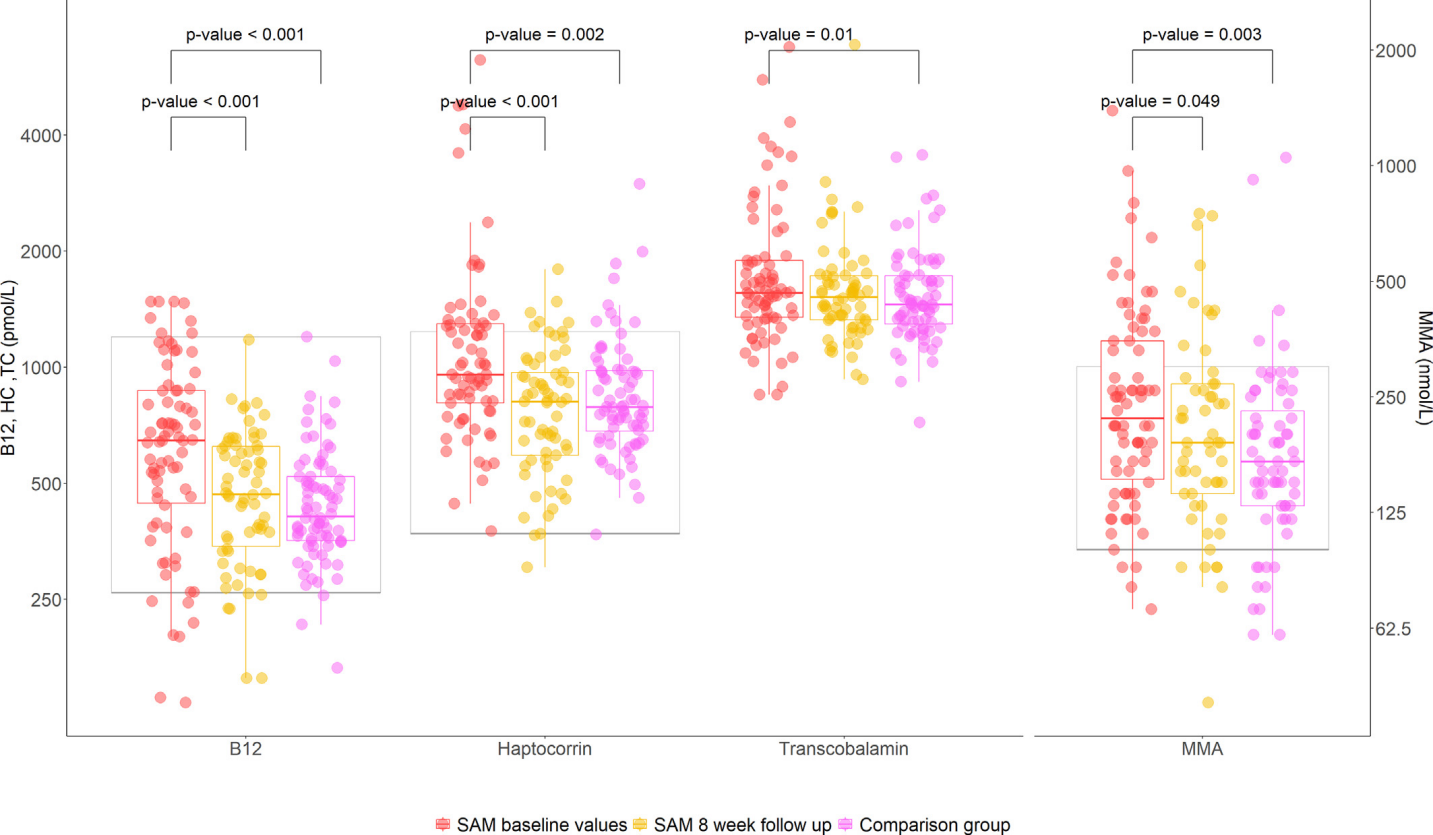
Biomarkers related to B12 in children with severe acute malnutrition (SAM) before and after treatment and in age- and sex-matched non-malnourished children. Data are shown on a logarithmic scale (colored dots) with medians and 25 to 75 percentiles (colored boxes). Reference levels are illustrated by grey squares. *P* values are shown for significant differences. Groups were compared by paired t-test (SAM before and after treatment) and linear regression models adjusted for age and sex (SAM compared with controls). B12 data were analyzed using Tobit regression due to right-censoring. MMA data were log transformed due to nonnormal distribution. HC, haptocorrin; MMA, methylmalonic acid; TC, transcobalamin.

The levels and distribution of vitamin B12-related biomarkers are presented in Figure 1, which displays the individual data points and the comparison between mean concentrations in children with SAM before and after nutritional treatment and the comparison with non-malnourished children. Plasma vitamin B12 was higher among children with SAM at baseline compared to controls (median 647 compared with 411 pmol/L; Figure 1). After 8 wk of SAM treatment, vitamin B12 decreased and was no longer higher than in the comparison group (median 469 pmol/L). MMA concentrations were higher in children with SAM compared to non-malnourished children (median 220 compared with 170 nmol/L) and decreased following treatment to reach concentrations comparable with those of the comparison group (median 190 nmol). Plasma HC in children with SAM was also higher at baseline compared to controls and decreased following treatment. At baseline, TC was significantly higher in SAM than in the comparison group, but there were no differences in TC concentrations in children with SAM before and after treatment. Median (IQR) and mean ± SD levels of all biomarkers in each group are described in Supplemental Table 2.

### Correlates of plasma B12

MMA was negatively correlated with vitamin B12 in children with SAM at baseline and follow-up, while HC was strongly positively correlated with vitamin B12 at baseline and follow-up in children with SAM and among non-malnourished children (Table 3). TC was not correlated with vitamin B12. At baseline, the mean vitamin B12 concentration in children with edema (*n* = 12) was marginally higher than that in malnourished children with no edema (222 pmol/L; 95% CI: −0.2, 444 pmol/L; *P* = 0.050). The highest concentrations of both baseline vitamin B12 and HC in the study was seen in a child with severe edema: ≥1480 pmol/L and 6257 pmol/L, respectively. This child had persisting edema during follow-up and continued to have high vitamin B12 levels compared to its peers after treatment (827 pmol/L, *P* = 0.03). In all other children with edemas at baseline, the edemas cleared during treatment. Children living with HIV (*n* = 7) also had higher baseline vitamin B12 concentrations than uninfected children with SAM (271.2 pmol/L; 95% CI: 15.8, 526.7; *P* = 0.04).

**TABLE 3:** Correlates of plasma B12 (pmol/L) in children with SAM and non-malnourished children.

	*n*	SAM baseline	*n*	SAM follow-up	*n*	No malnutrition
		Mean (95% CI), *P*		Mean (95% CI), *P*		Mean (95% CI), *P*
**MMA**, nmol/L	75	−0.56 (−0.93, −0.19), *P* = 0.003	54	−0.35 (−0.62, −0.08), *P* = 0.01	65	−0.22 (−0.46, 0.02), *P* = 0.08
**HC**, pmol/L	72	0.24 (0.16, 0.32), *P* < 0.001	62	0.34 (0.21, 0.47), *P* < 0.001	75	0.34 (0.27, 0.40), *P* < 0.001
**TC**, pmol/L	74	0.02 (−0.07, 0.10), *P* = 0.67	63	0.01 (−0.05, 0.06), *P* = 0.84	74	0.02 (−0.06, 0.10), *P* = 0.57
**Edema** (at baseline)						
No edema	66	ref	65	ref	80	NA
Edema	12	222 (−0.2, 444), *P* = 0.050	12	12 (−110, 133), *P* = 0.85	0	NA
**HIV status** (at baseline)						
HIV negative	43	ref	35	ref	0	NA
HIV positive	7	271.2 (15.8, 526.7), *P* = 0.04	6	−86.2 (227.4, 55.1), *P* = 0.23	0	NA

Abbreviations: CI, confidence interval; HC, haptocorrin; MMA, methylmalonic acid; NA, not applicable; SAM, severe acute malnutrition; ref, referent; TC, transcobalamin.

Estimates are based on Tobit regression with right-censoring at 1480 pmol/L adjusted for age and sex.

## Discussion

In this study, we initially found unexpectedly high plasma vitamin B12 in children with SAM, especially in those with nutritional edema and HIV. This motivated us to further investigate vitamin B12 status by measuring MMA, which increases as functional vitamin B12 sufficiency decreases, and by measuring 2 vitamin B12 binding proteins in plasma, HC and TC. In contrast to what the vitamin B12 concentrations suggested, MMA concentrations in children with SAM indicated lower vitamin B12 function than in a non-malnourished comparison group at baseline and improvement after treatment. The vitamin B12 binding protein HC was strongly correlated with plasma vitamin B12. HC was also elevated in children with SAM and decreased after treatment, in line with plasma vitamin B12. TC in children with SAM did not change after treatment, but baseline concentrations were higher in children with SAM than in the comparison group.

Contrary to many other micronutrients, plasma vitamin B12 concentrations are not affected by inflammation, and the direct measurement is relatively inexpensive and widely available [3]. Although it may not be considered the gold standard, plasma vitamin B12 is therefore the most commonly used biomarker of vitamin B12 status, and it is generally regarded as reliable to reflect long-term vitamin B12 status at population levels [3]. However, direct measurement of serum or plasma vitamin B12 does not consider variations in the vitamin B12 binding proteins TC and HC and may therefore lead to misinterpretation.

In our study, we found that baseline HC was strongly correlated with vitamin B12, pointing to elevated HC as a possible explanation for the high plasma vitamin B12 observed in children with SAM. Previous studies have reported elevated HC associated with higher-than-normal vitamin B12 levels in various health conditions, for example, related to liver or hematological diseases [11,18]. However, to our knowledge, there are only a few reports of this phenomenon in populations with malnutrition. A study [19] from Nigeria from 1983 also found high levels of both vitamin B12 and HC in acutely malnourished children but used a different method because direct measurement of total HC was not possible at the time. The authors measured unsaturated HC and found that elevated levels were associated with high vitamin B12, which likely reflects the same link between HC and vitamin B12 in malnutrition as in our study. In parallel with our findings, the 1983 study found that high levels of both vitamin B12 and unsaturated HC were more pronounced in children with edematous malnutrition. In our SAM population, we observed higher plasma vitamin B12 in children with edema. In particular, one child with severe edema had very high baseline concentrations of both vitamin B12 and HC, and that same child had persistent edema at follow-up and continued to have elevated vitamin B12 concentration compared to its peers. Our observations and the early study from Nigeria indicate a potential interaction between edematous malnutrition and elevated vitamin B12 and HC, which merits further research.

A possible link between elevated vitamin B12 and edematous malnutrition may be found in hepatic metabolic pathways. It has been suggested that the hallmark hepatic steatosis of edematous malnutrition may be caused by disturbed hepatic metabolism due to a lack of vital nutrients [20], and recently, it has been discussed whether deficient intake of the nutrient choline is implicated in this mechanism in children with edematous SAM [21]. In a study in women with anorexia nervosa, the authors found higher levels of vitamin B12 associated with lower BMI and also suggested choline deficiency as a potential underlying cause of their findings [22]. This could be explained by a compensatory mobilization of choline from peripheral organs, which could potentially increase levels of other metabolites in the one-carbon metabolism pathway, including vitamin B12. However, this study did not include measurement of vitamin B12 binding proteins, and although elevated HC has been linked to various illnesses affecting the liver [11], further studies are needed to elucidate the potential connection between edematous malnutrition, choline deficiency, hepatic metabolism, and HC. Our results also revealed temporarily higher concentrations of vitamin B12 in children with HIV and SAM. Elevated vitamin B12 in HIV-infected has been previously reported, but although altered vitamin B12 metabolism in HIV has been suggested, to our knowledge, no pathogenic explanation has been identified [11,18]. Only 7 children in our sample were HIV-positive, but larger samples may reveal more about the possible interaction between malnutrition, HIV, and vitamin B12.

In contrast to the other biomarkers of vitamin B12, TC levels did not decrease after treatment among children with SAM, although TC at baseline was higher in children with SAM than in the comparison group. There was no correlation between baseline TC and vitamin B12. We refrained from defining a normal range for TC because the only available reference material was based on an adult population [14], and TC is likely to be age-dependent. A 1982 study from The Democratic Republic of the Congo (then Zaïre) found significantly higher TC in children than in adults [23], whereas a study from Nigeria in the same year conversely suggested that TC would increase with age [24]. More recent data from a Danish cohort measured TC levels at 9 mo of age, which were similar in range to our results [25]. However, both TC and HC may vary greatly across different populations due to genetic variation [24]. Thus, the use of TC as a biomarker in children may be limited until relevant reference data are available.

Unlike plasma vitamin B12, measurement of MMA is unaffected by increases in vitamin B12 binding proteins. Although it may be elevated independently of vitamin B12 in individuals with renal impairment or gut dysbiosis, it is considered the most sensitive marker of vitamin B12 function [3]. We were therefore able to estimate the burden of vitamin B12 deficiency in our cohort more accurately based on this biomarker. Although measurements of plasma MMA showed higher prevalence of vitamin B12 insufficiency in SAM than in non-malnourished children, only 23% of children with SAM had MMA concentrations suggesting reduced vitamin B12 function. This prevalence of vitamin B12 deficiency is notably less than that in former studies in children with acute malnutrition [6,9]. However, in a cross-sectional study of micronutrient status among children with stunting in Uganda, only 15.8% of the population had elevated levels of MMA, which is similar to our findings [26]. Authors attributed this relatively small proportion to higher fish consumption near Lake Victoria, which is also likely to influence the results from our cohort. These potential regional differences make the identification of reliable and available biomarkers of vitamin B12 status all the more important, as the need for supplementation may vary. Other studies in children with acute malnutrition have found mostly normal vitamin B12 levels, but since many of these have been based on direct measurement of plasma vitamin B12, considering our findings, their results may have underestimated the prevalence of vitamin B12 deficiency in these populations [[27], [28], [29]].

Our study design, combining a longitudinal study of children with SAM with observations from sex-, age-, and community-matched controls, provided a strong basis for assessing vitamin B12 status in both malnourished and non-malnourished Tanzanian children. Including analyses of MMA and the vitamin B12 binding proteins TC and HC allowed us to provide an explanation for the finding of elevated plasma vitamin B12 in children with SAM. However, some important limitations are worth mentioning. Although the sample size of the study was adequate overall, the subgroup with edematous malnutrition was too small to draw strong conclusions. Additionally, there was a significant loss to follow-up, which may have introduced selection bias in our analyses. Although our analyses of transport proteins provide a plausible pathway for the elevated vitamin B12 levels in SAM in our cohort, other important confounders, which we were not able to include due to data limitations, may have influenced the findings. Breastfeeding practices, including exclusive breastfeeding and breastfeeding beyond 1 y, have been shown in some contexts to correlate with lower vitamin B12 levels in both healthy and malnourished children [9,25,26]. These practices may be unevenly distributed between malnourished and non-malnourished children. Likewise, MMA, although considered a reliable marker of vitamin B12 function, may be elevated in children with renal failure and altered gut microbiome, conditions which may be overrepresented in children with SAM compared to non-malnourished children. Thus, future studies may benefit from including data on breastfeeding, renal function, and the gut microbiome. Due to limitations in sample material, we were unable to measure the part of TC saturated with vitamin B12 (holoTC, active vitamin B12), which has been suggested as a biomarker to mirror vitamin B12 status better than direct measurement of vitamin B12 itself, and in addition, is not influenced by variations in HC [30]. Thus, future studies in children with malnutrition may consider using holoTC instead of vitamin B12. Homocysteine, another marker of vitamin B12 function, may also provide additional insights. Finally, although HC was identified as a plausible driver of high vitamin B12, we were unable to draw conclusions regarding the cause of elevated HC and the possible link with edematous malnutrition. Longitudinal studies in larger cohorts or in settings where edematous SAM is more prevalent that include assessments of hepatic metabolism and steatosis as well as vitamin B12, MMA, and HC may provide further insight.

In conclusion, in this study, we initially found high plasma vitamin B12 in children with SAM, especially in those with edema, which decreased during treatment. However, subsequent analysis of plasma MMA, a marker of vitamin B12 deficiency, showed that children with SAM had poor vitamin B12 status, which improved with treatment. Our findings indicate that direct measurement of vitamin B12 may not be appropriate as a stand-alone biomarker of vitamin B12 status in children with SAM, and alternative biomarkers such as MMA and holoTC should be explored in this population. In our population, the discrepancy between estimates of vitamin B12 status based on plasma vitamin B12 and MMA seemed to be attributed to elevations of the binding protein HC. Further studies are needed to understand the possible link between elevated HC and edematous malnutrition, and the possible role of the liver.

## Author contributions

The authors’ responsibilities were as follows – MFO, HF, EN: designed the research, conceived the project, developed the overall research plan, and were responsible for study oversight; GP, HK, MA, BK: conducted the research; MFO, RM, EN: analyzed data; RM, MFO, EN, MG, HF, SF, AB: wrote the paper; MFO: had primary responsibility for final content; and all authors: read and approved the final manuscript.

## Data availability

Data cannot be shared publicly because Tanzanian ethics guidelines restrict any data sharing, including deidentified data, without the approval of the Medical Research Coordinating Committee (MRCC). Data are available from the National Institute for Medical Research and can be shared with researchers who meet the criteria for access to confidential data only after completing a data transfer agreement and approval by the MRCC. The MRCC can be contacted at ethics@nimr.or.tz.

## Funding

This research was funded by the Department of Health and Social Care (DHSC), the Foreign, Commonwealth & Development Office (FCDO), the Medical Research Council (MRC), and Wellcome, grant reference: MR/T003731/1. The funder had no role in the study design or interpretation of results.

## Conflict of interest

The authors report no conflicts of interest.
