# Supplementary material for: Plasma Vitamin B12 May Be a Misleading Biomarker among Children with Severe Acute Malnutrition: An Observation from Mwanza, Tanzania
Source: J Nutr. 2025 May 16;155(9):2898–905. doi: 10.1016/j.tjnut.2025.04.033 (PMC12799436; doi:10.1016/j.tjnut.2025.04.033)
Supplement: multimedia component 1 [file mmc1.docx]

| **Supplementary Table 1:** Nutritional composition of trial RUTF | |
| --- | --- |
| **Nutrient** | **Mean value/100g** |
| *Macronutrients* | |
| Energy, kcal | 543 |
| Protein, g | 14.1 |
| Protein, dairy, g | 0.84 |
| Lipids, g | 33.8 |
| Linoleic acid, g | 3.7 |
| alpha linoleic acid, g | 1.02 |
| Carbohydrates, mg | 44.5 |
| Total fibres | 1.6 |
| Moisture, g | 0 |
| DHA, mg | 0 |
| EPA, mg | 0 |
| *Micronutrients* | |
| Phosphorus, mg | 384 |
| of which free phosphorus, mg | 340 |
| Calcium, mg | 358 |
| Potassium, mg | 1374 |
| Magnesium, mg | 88 |
| Zinc, mg | 12.8 |
| Copper, mg | 1.7 |
| Iron, mg | 11.4 |
| Iodine, µg | 123 |
| Selenium, µg | 38 |
| Sodium, mg | 166 |
| Vitamin A, mg RE | 0.86 |
| Vitamin D, µg | 15 |
| Vitamin E, mg alpha-TE | 20 |
| Vitamin C, mg | 50 |
| Vitamin B1, mg | 0.54 |
| Vitamin B2, mg | 1.6 |
| Vitamin B6, mg | 0.6 |
| Vitamin B12, µg | 1.6 |
| Vitamin K, µg | 28 |
| Biotin, µg | 64 |
| Folic acid, µg | 200 |
| Pantothenic acid, mg | 3 |
| Niacin, mg | 5 |
| **Ingredients:** Milk powder (non-fat, dry); Milk (sweet whey powder); Palm oil; Canola oil; Sugar; High-oleic peanuts; Standard high ꞷ-6 peanuts; Perilla oil; Hydrogenated vegetable oil; Micronutrient powder. RUTF = Ready-to-use therapeutic food, DHA = docosahexaenoic acid, EPA = eicosapentaenoic acid | |

# **Supplementary material: “Plasma B12 may be a misleading biomarker among children with severe acute malnutrition: An observation from Mwanza, Tanzania” – Rikke Møller *et al.***

| **Supplementary Table 2:** Plasma B12, MMA, HC and TC among children with SAM and non-malnourished controls | | | | | | | | | |  |
| --- | --- | --- | --- | --- | --- | --- | --- | --- | --- | --- |
|  | **SAM baseline** | | | **SAM 8 weeks** | | | **No malnutrition** | | | |
|  | n | Median (IQR) | Mean (sd) | n | Median (IQR) | Mean (sd) | n | Median (IQR) | Mean (sd) | |
| **B12**, pmol/L | 78 | 647 (444; 870) | 690 (350) | 66 | 469 (343; 622) | 490 (189) | 80 | 411 (355; 521) | 461 (173) | |
| **MMA**, nmol/L | 78 | 220 (153; 350) | 284 (212) | 56 | 190 (140; 270) | 239 (159) | 67 | 170 (130; 230) | 203 (157) | |
| **HC**, pmol/L | 73 | 959 (808; 1297) | 1283 (1032) | 65 | 815 (591; 969) | 819 (305) | 76 | 789 (682; 981) | 898 (386) | |
| **TC**, pmol/L | 76 | 1560 (1346; 1890) | 1884 (1021) | 66 | 1519 (1327; 1726) | 1660 (778) | 75 | 1455 (1294; 1725) | 1592 (509) | |
| MMA = methylmalonic acid, HC = haptocorrin, TC = transcobalamin, SAM = severe acute malnutrition. No malnutrition = mid upper arm circumference (MUAC) >125 and weight-for-height z-score (WHZ)>-2. | | | | | | | | | |  |
